# Stable Anxiety and Depression Trajectories in Late Adolescence for Oral Contraceptive Users

**DOI:** 10.3389/fpsyt.2022.799470

**Published:** 2022-05-23

**Authors:** Anne Marieke Doornweerd, Susan Branje, Stefanie A. Nelemans, Wim H. J. Meeus, Estrella R. Montoya, Iris M. Engelhard, Joke M. P. Baas, Lotte Gerritsen

**Affiliations:** ^1^Department of Experimental Psychology, Utrecht University, Utrecht, Netherlands; ^2^Department of Clinical Psychology, Utrecht University, Utrecht, Netherlands; ^3^Department of Youth and Family, Utrecht University, Utrecht, Netherlands; ^4^Huijs GGZ, Den Bosch, Netherlands

**Keywords:** development, adolescence, anxiety, depression, oral contraceptives (OCs)

## Abstract

**Background:**

The use of oral contraceptives (OCs) has been associated with increased incidences of anxiety and depression, for which adolescents seem to be particularly vulnerable. Rather than looking at singular outcomes, we examined whether OC use is associated with depressive and anxiety symptom trajectories from early adolescence into early adulthood.

**Materials and Methods:**

Data from 178 girls were drawn from the Research on Adolescent Development and Relationships (RADAR-Y) younger cohort study. We used assessments on 9 waves from age 13 until 24. Developmental trajectories of ratings on the Reynolds Adolescent Depression Scale (RADS-2) and the Screen for Child Anxiety Related Emotional Disorders (SCARED) were compared between never and ever users of OCs.

**Results:**

Never users showed increases in depressive and anxiety symptoms in late adolescence, whereas OC users showed a stable level of symptoms throughout adolescence. This effect remained after adjusting for baseline differences between groups in romantic relationships, sexual debut, educational level, smoking, drinking, and drug use. Age of OC use onset did not significantly predict symptom development.

**Conclusions:**

OC use in adolescence was related to an altered developmental trajectory of internalizing symptoms, in which OC users did not show an increase in depressive and anxiety symptoms in late adolescence, whereas never users did. The question remains whether this altered symptom trajectory can be considered a protective effect of OC use on psychopathology. Additional research is needed to improve our understanding of the long-term consequences of OC use on mental health.

## Introduction

The oral contraceptive pill (OC; “the pill”) has been on the market for half a century, is used by about 100 million women world-wide, and is one of the most extensively studied pills in the history of medicine (1, 2). In countries such as the Netherlands, about one in five adolescent girls use OCs to prevent pregnancy, alleviate dysmenorrhea, and/or treat acne (3, 4). Whereas, the physical side effects of OCs have been subject to a considerable amount of research, a surprising lack of knowledge exists about its effects on affect, including effects on affect-related brain function (5–7). Some studies suggest that women taking OCs will not be subjected to adverse emotional changes (8, 9), or might even experience a protective effect on their mental health (10–14). However, in recent large observational studies OC use has been associated with higher depressive symptoms (15–17), use of antidepressants (17, 18), and an increased risk of suicide (19). Evidence regarding OCs and anxiety has been limited, with some studies showing that OC use is associated with the onset of anxiety symptoms (20, 21), and lower efficacy of exposure-based therapy (22, 23).

The mixed findings have raised doubts about whether the association of OC use with mood deterioration is due to hormonal disruption. The OC pill contains synthetic versions of the hormones estradiol (E) and progesterone (P) and suppresses natural gonadal hormone production (2). Since natural fluctuations of E and P have been associated with emotion regulatory functioning and mood, OC use may result in emotion dysregulation and internalizing symptoms (6). On the other hand, the different outcomes could merely result from existing and unmeasured group differences between OC users and non-users that make OC users inherently more at risk of psychopathology beyond any influence of OC hormones (24). For instance, behavioral and social factors, such as earlier sexual debut, age at menarche, and smoking, have been identified as confounders for both OC use and depression (8, 25, 26).

Additionally, the conflicting evidence may be especially prominent in studies of only adult OC users, as studies including information on adolescent OC use find adolescents to be particularly vulnerable to developing mood symptoms and disorders after OC use (15–17, 27). This finding may be explained by the absence of the survivor bias effect in first time adolescent OC users compared to adult women with possible previous OC experiences. Then again, adolescent OC users may also be more susceptible to the change in hormonal milieu after starting OCs. Adolescence is considered to be a vulnerable period for the development of depression and anxiety disorders (28–30), with girls being twice as likely to experience depressive and anxiety symptoms compared to boys (31, 32). During this developmental period, the gonadal hormones are at the forefront of brain development (33), and hormonal disruptions are therefore likely to affect brain development and mental health in the long run (34). Given that adolescent girls use OCs at an increasingly younger age and for purposes beyond contraception (3), it is important to gain more insight into the effects of OC's on mental health in adolescence.

Whereas depression in adolescents taking OCs has been the focus in some previous longitudinal studies, the effect of adolescent OC use was mostly assessed retrospectively. Therefore, the present study aimed to examine OC's effect on both depression and anxiety symptoms throughout adolescence. Our study also goes beyond prior research by modeling OC effects on the depressive and anxiety symptom trajectories from early adolescence into early adulthood. These symptom trajectories may be more sensitive to OCs effect than assessing clinical diagnoses and differences in singular outcomes. This allows examining the effect of OC use on symptom development over time while taking the naturally fluctuating development of internalizing symptoms into account. Data were used from a longitudinal data set in the Netherlands on adolescent development. Whereas, baseline information before OC onset is often unavailable in prior work, the current dataset allowed us to include measurements on mental health and adolescent developmental markers over the course of several years before and after OC onset. We used this information to a) examine the relationship between OC use and depression and anxiety symptom development throughout adolescence and into early adulthood, b) explore the effect of age of OC onset on these symptom trajectories, and c) identify possible confounders and risk factors in adolescent development of an effect of OC use on internalizing symptoms.

## Materials and Methods

### Participants and Procedure

Participants were drawn from the ongoing Research on Adolescent Development and Relationships (RADAR-Y) young population cohort study (35). They were recruited in schools in the province of Utrecht and four cities the Netherlands in 2006. After the first assessment, participants we used in the present study were followed up for nine measurement waves up to 2017. At the first assessment, 214 adolescent girls were included with a mean age of 13.01 years (SD = 0.44). Until age 18, measurements were done annually; afterwards (after wave 6) they were done biannually. From this sample only participants were included who provided information on oral contraceptive use. Thirty-five participants had missing data on contraceptive use. Four participants used other forms of hormonal contraceptives (e.g., injection or intrauterine device), of which 3 girls had used OCs prior to the current alternative contraceptive. The final sample consisted of 178 native Dutch girls, with a mean age of 12.99 (SD = 0.43) at Wave 1 and 23.83 (SD = 0.43) at Wave 9. Most participants had a medium or high socioeconomic status (SES) (85.4%).

### Measures

#### Oral Contraceptive Use

Current and past use of OC was determined via self-report at Wave 6 with the question: “which (hormonal) contraceptive do you currently use?” Participants could indicate when the first intake of their current contraceptive occurred [in months and years, which previous (other) contraceptive they had used, and which dates they started and stopped (in months and years)]. No information was available on OC type. Girls who reported no previous or current OC use were defined as never users of OCs. Ever users of OCs were defined as girls who reported to use OCs currently or previously. Follow up analyses used age of OC onset (*n* = 137), which was determined by age, date of assessment, and date of onset OC use. Early OC users were defined as users with first intake of OCs before the age of 15 and late OC users were defined as starting OC use at age 15 or older. The age of 15 was selected as a cutoff to separate early and middle/late adolescence into groups comparable in size (*n* = 33 for early users, *n* = 44 for late users).

#### Depression

Depressive symptoms were assessed by the Dutch adjusted version of the Reynolds Adolescent Depression Scale, 2^nd^ ed. (RADS-2) (36). The RADS-2 is a self-report measure developed to measure cognitive, motor, somatic and interpersonal depressive symptoms in adolescents. The scale consists of 23 items (the anhedonia subscale was not administered on Waves 2-6) on a 4-point Likert scale ranging from 1 (almost never) to 4 (most of the time), with a minimum score of 23 and maximum score of 92. Items are part of the subscales dysphoric mood, negative self-evaluation, and somatic complaints. In this study, the scale showed good internal consistency across all waves, with a Cronbach's alpha of 0.93. A previous study has shown good psychometric properties for the RADS-2 in adolescents (37).

#### Anxiety

Anxiety symptoms were assessed with the Screen for Child Anxiety Related Emotional Disorders (SCARED) (38). The SCARED is a self-report instrument designed to measure anxiety symptoms in children and adolescents. The SCARED consists of 38 items scored on a 3-point Likert scale ranging from 1 (almost never) to 3 (often), with a minimum score of 38 and maximum score of 114. The scale assesses the subscales somatic/panic, general anxiety, separation anxiety, social phobia, and school phobia. The internal consistency for the SCARED was good across all waves, with a Cronbach's alpha of 0.93. Multiple previous studies have shown the SCARED has good psychometric properties (38–40).

#### Covariates

First, covariates were selected if they had previously been associated with OC use and/or internalizing symptoms: age at menarche (25), (age at) sexual debut [yes/no] (25), romantic relationships [yes, no] (41), smoking history [no, yes] (26), religion [not religious, religious] (42), alcohol use [0-5, no alcohol this past month to alcohol every day this month] (43), hard drug use [no, yes] (44), childhood trauma [Childhood Trauma Questionnaire (CTQ)] (45), neuroticism [BIG5] (46), educational level [wave 3] (47), and SES [low, middle/high] (48). Next, the variables that were significantly different between never and ever OC users (or never, early, and late users) were included in the analyses (Table 1). These were sexual debut (*p* < 0.001), romantic relationships (*p* < 0.001), education (*p* = 0.006), religion (*p* = 0.045), alcohol use (*p* = 0.049), smoking history (*p* = 0.033) and drug use (*p* = 0.082 for never/ever, *p* = 0.040 for never/early/late).

**Table 1:** Study sample characteristics depending on history of oral contraceptive use.

**Characteristic**	**Never users (*n* = 60)**	**Ever users (*n* = 118)**	***P***	**Early users (*n* = 33)**	**Late users (*n* = 44)**	***P***
Age, mean (SD)	12.9 (0.4)	13.0 (0.5)	0.086	13.0 (0.4)	13.0 (0.5)	0.975
Age at menarche, mean (SD)	12.5 (1.4)	12.5 (1.4)	0.187	12.1 (1.5)	12.7 (1.5)	0.142
Age at sexual debut, mean (SD)	14.7 (4.4)	15.1 (2.1)	0.537	15.0 (1.2)	15.8 (3.0)	0.186
Sexual debut, N(%)	12/60 (20)	93/115 (80.9)	<0.001	28/33 (84.8)	33/41 (80.5)	0.624
Romantic Relationships, N(%)	32/56 (57.7)	93/102 (91.2)	<0.001	24/27 (88.9)	35/40 (87.5)	0.863
Education, mean (SD)	6.4 (2.2)	5.3 (2.5)	0.006	5.2 (2.7)	5.9 (2.4)	0.297
Low Family SES, N(%)	7/59 (11.7)	17/117 (14.4)	0.627	3/33 (9.1)	4/44 (57.1)	1.00
Religious, N(%)	37/60 (61.7)	54/118 (45.8)	0.045	11/33 (33.3)	24/44 (54.5)	0.064
Smoking history, N(%)	18/60 (30.0)	55/118 (46.6)	0.033	17/33 (51.5)	14/44 (31.8)	0.081
Alcohol Use, mean (SD)	1.1 (0.9)	1.4 (0.8)	0.049	1.4 (1.0)	1.3 (0.7)	0.613
Drug Use, N(%)	11/60 (18.3)	36/118 (30.5)	0.082	14/33 (42.4)	14/44 (31.8)	0.338
Childhood Trauma, mean (SD)	1.3 (0.3)	1.3 (0.3)	0.985	1.3 (0.3)	1.3 (0.3)	0.913
Neuroticism^a^, mean (SD)	4.3 (1.17)	4.3 (1.22)	0.928	4.4 (1.1)	4.4 (1.2)	0.987

*^a^Scores reflect emotional stability with lower scores meaning higher levels of neuroticism*.

### Analyses

Descriptive statistics were computed for all OC groups (never users, ever users, early users and late users). Growth curve models were used to describe the effect of OC use on the developmental trajectories of both depressive and anxiety symptoms (49). First, a best-fit model was determined that best captured the observed symptom changes in different developmental periods for both depressive and anxiety symptoms. This was done by comparing the chi-square difference (Δχ^2^) in log likelihood (-2LL) of a one-slope model, two slope model or three slope model with age centered by subtracting 16 from the original values (to compare slopes for early and late adolescence). The one-slope model assumed a linear relationship between age and anxiety or depressive symptoms, where the two-slope model fitted two slopes before and after age 16. The three-slope model plotted one slope for early adolescence (<14 years), one slope for middle to late adolescence (14–18 years), and one slope for young adulthood (> 18 years). The best fitting model used two age slopes to model the development of symptoms from age 13–24.

The final growth curve model assessed within-subject variation using a random intercept and the two age slopes. In the crude models, between-subject variation was modeled by fixed effects for OC use [never/ever]. The age slopes and two-way interaction effects of OC use and both slopes were included to estimate whether the course of depressive and anxiety symptoms differed according to OC use. The adjusted models included the covariates romantic relationships, sexual debut, educational level, religion, alcohol use, drug use, and smoking history as fixed effects. This resulted in two crude and two adjusted models for OC use effects on depressive and anxiety symptoms, respectively. All growth curve analyses were performed using PROC MIXED in SAS 9.4 (SAS Inc, Cary, NC).

Follow-up analyses grouped OC use as never, early (<15 years), or late (≥ 15 years) users to examine the effect of age of OC onset on mood development. Additionally, sensitivity analyses were conducted by restructuring the data using age of OC onset in a 2-slope model, setting the slopes to before and after OC onset (slopes before and after the mean age of OC onset for never users). Modeling the effect of OC use based on age of OC onset rather than centered age resulted in similar main results (see Supplementary Table 1 and Supplementary Figure 1). The main growth curve models that used age to model depressive and anxiety symptoms had more power than models using OC onset, because 41 participants had missing data for date of OC onset.

## Results

Of the total sample of 178 girls, 60 reported they have never used hormonal contraceptives before and 118 reported they have used OCs. Of the OC users, 6 girls were not currently using OCs but had used them in the past, and the other 112 were currently using OCs. For 77 girls, the age of OC use onset could be determined; the other 41 girls had missing data for date of OC onset. The mean age of OC onset was 14.9 (SD = 1.6). Of the OC users, 33 were considered early users and 44 considered late users. Table 1 displays the descriptive statistics for never and ever users of OC and for early and late users. OC users were more likely to have had romantic relationships and their sexual debut. Additionally, OC users had a lower level of education, and were more likely to have smoked, have drunk alcohol and be non-religious compared to never users. In Supplementary Table 2 the correlations between the descriptive characteristics and internalizing symptoms are listed. Table 2 describes the means and standard deviations of depressive and anxiety symptoms of the final sample at each wave. Supplementary Table 3 lists the correlations between anxiety and depression for each wave.

**Table 2:** Depressive and anxiety symptoms per study wave.

	**Depressive symptoms M(SD)**			**Anxiety symptoms M(SD)**		
**Wave**	**All**	**Never users**	**Ever users**	**All**	**Never users**	**Ever users**
Age 13 (W1)	39.5 (11.9)	38 (11.7)	40.3 (12)	53.6 (10.4)	54.2 (11.7)	53.3 (9.5)
Age 14 (W2)	38.2 (13.1)	36.8 (14.2)	39 (12.5)	52.2 (12.4)	53.9 (14.5)	51.2 (11)
Age 15 (W3)	40.3 (14.6)	37.9 (14)	41.6 (14.8)	53.5 (13.4)	52.4 (13.7)	54.1 (13.3)
Age 16 (W4)	40.3 (14.3)	38.9 (14.9)	41.1 (14)	52.5 (13.5)	52.7 (14.7)	52.5 (12.9)
Age 17 (W5)	39.1 (13.9)	38.4 (13.6)	39.6 (14)	51.6 (11.8)	51.1 (11.9)	51.9 (11.7)
Age 18 (W6)	39.9 (13.9)	40.3 (14.4)	39.7 (13.7)	52.2 (12.6)	52.5 (13.9)	52 (11.9)
Age 20 (W7)	40.0 (11.9)	41.6 (13.2)	38.7 (10.7)	52.9 (10.7)	54.4 (12.5)	51.7 (9)
Age 22 (W8)	41.4 (13.4)	44.9 (15.7)	38.4 (10.4)	53.2 (11.4)	55 (13.5)	51.5 (9)
Age 24 (W9)	42.6 (13.2)	47.5 (15.1)	38.7 (10.1)	53.1 (12.3)	56.9 (15.3)	50.2 (8.2)

### Anxiety and Depressive Symptom Trajectories

The best fit model that was used to determine the effect of OC use on symptom development throughout adolescence modeled symptom trajectories over two slopes before and after 16 years for both depression [χ^2^(6) = 875.9, AIC = 9997.2, BIC = 10020.7] and anxiety [χ^2^(6) = 846.9, AIC = 9010.2, BIC = 9033.7]. For the depressive symptoms, there was no significant main effect of slope 1 (ß = 0.02, *p* = 0.0.947). For the whole sample, depressive symptoms significantly increased in late adolescence (ß = 0.38, *p* = 0.027).

The development of anxiety symptoms in adolescence did not show significant main effects of slopes. Anxiety symptoms remained stable in early (ß = −0.43, *p* = 0.147) and late adolescence (ß = 0.08, *p* = 0.613).

### OC Use and Anxiety and Depressive Symptoms

**Figure 1:**
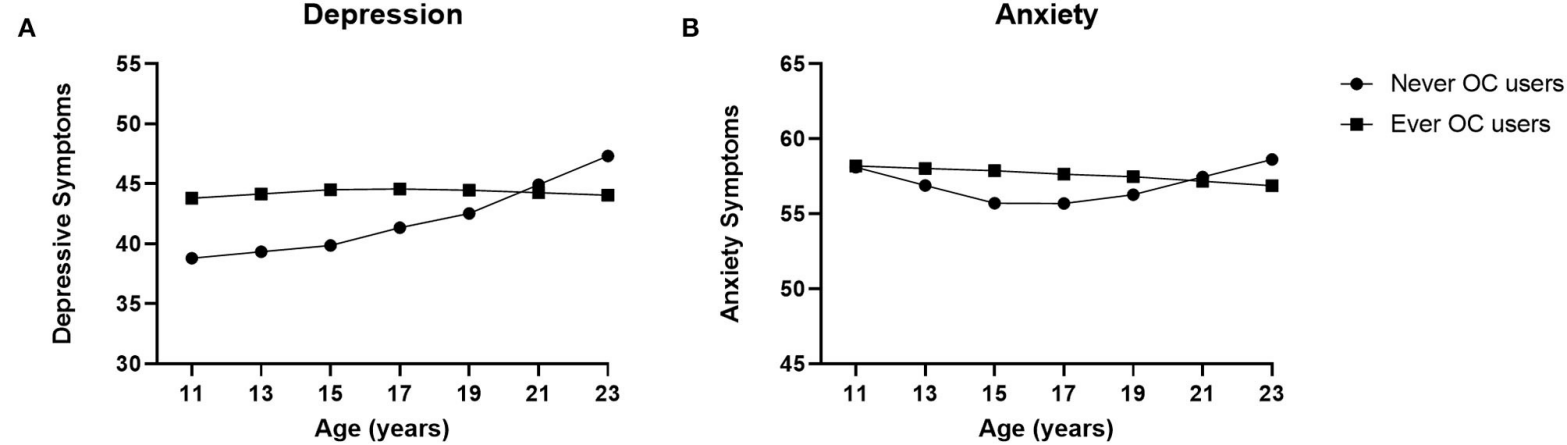
Oral Contraceptive (OC) use and anxiety and depression modeled symptom trajectories. Growth curve models for the depressive and anxiety symptoms for never users of oral contraceptives (OCs) and ever users of OCs. **(A)** In late adolescence depression scores increased significantly for never users, whereas OC users showed stable depression scores throughout adolescence. **(B)** An increase in anxiety symptoms toward late adolescence was significant only for never users of OCs, with stable levels of anxiety symptoms for OC users. Means are adjusted for romantic relationships, sexual debut, education level, religion, smoking history, alcohol use, and drug use.

The trajectories over time for depression and anxiety are shown in Figure 1. Table 3 shows the model fit, standardized regression coefficients and standard errors of the models. Depressive symptoms showed an overall increase in late adolescence with a significant main effect of slope 2 (*p* = 0.003). There was a significant interaction effect of slope 2 and OC use (*p* < 0.001): never users of OC showed an increase in depressive symptoms in late adolescence, whereas OC users showed a stable trajectory of depressive symptoms throughout adolescence and young adulthood. The model adjusted for covariates showed similar results, with a significant main effect of age slope 2 (*p* = 0.001) and a significant interaction effect of age slope 2 and OC use (*p* < 0.001), indicating a general increase in depressive symptoms in late adolescence for girls and women who have never used OCs.

**Table 3:** Model fit, standardized regression coefficients and standard errors of fixed and random effects as predictors of development of depressive and anxiety symptoms with never and ever users of oral contraceptives.

**Parameters**	**Estimate**	**SE**	**χ^2^**	**df**	**AIC**	**BIC**
**Depression - crude**
Intercept	38.11	1.76	815.64	6	8868.6	8890.9
Age 1	0.22	0.46				
Age 2	1.11*	0.25				
OC use	2.41	2.41				
OC use*age 1	−0.11	0.58				
OC use*age 2	−1.25**	0.33				
**Depression - adjusted**
Intercept	39.09	3.68	700.41	6	8067.0	8088.2
Age 1	0.27	0.49				
Age 2	1.20*	0.26				
OC use	4.54	2.56				
OC use*age 1	−0.09	0.61				
OC use*age 2	−1.30**	0.34				
Romantic relationships	−4.23	2.53				
Sexual debut	−0.06	2.32				
Educational level	0.51	0.34				
Religion	−1.48	1.67				
Alcohol use	−3.06*	1.05				
Smoking	4.20*	1.85				
Drug use	5.18*	2.05				
**Anxiety - crude**
Intercept	51.85	1.65	785.84	6	8098.4	8120.7
Age 1	−0.64	0.51				
Age 2	0.47	0.25				
OC use	0.89	2.04				
OC use*age 1	0.56	0.64				
OC use*age 2	−0.74	0.33				
**Anxiety - adjusted**
Intercept	55.11	3.42	692.46	6	7339.8	7361.1
Age 1	−0.60	0.53				
Age 2	0.59	0.24				
OC use	2.69	2.32				
OC use*age 1	0.52	0.68				
OC use*age 2	−0.74*	0.32				
Romantic relationships	−4.66	2.38				
Sexual debut	−0.05	2.20				
Educational level	0.21	0.32				
Religion	−0.72	1.58				
Alcohol use	−3.10*	0.99				
Smoking	2.39	1.75				
Drug use	5.93*	1.94				

*p <0.05, **p <0.001; Adjusted models are corrected for educational level, religion, smoking history, alcohol use and drug use; SE, standard error; df, degrees of freedom. AIC, Akaike Information Criterion; BIC, Bayesian Information Criterion.

In the crude model for anxiety symptoms, only the interaction effect between slope 2 and OC use was significant (*p* = 0.023). Again, an increase toward the end of adolescence and young adulthood was seen only for never users of OCs (Table 2). OC users showed a slight decrease of anxiety symptoms in late adolescence. Adjusting for the covariates showed similar results, with a significant interaction effect of age slope 2 and OC use (*p* = 0.020).

### Age of OC Use and Anxiety and Depressive Symptoms

**Figure 2:**
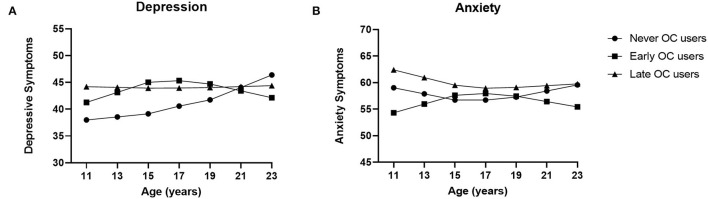
Oral Contraceptive (OC) use, age of onset, and anxiety and depression modeled symptom trajectories. Growth curve models for the depressive and anxiety symptoms for never, early (<15 years), and late (≥15 years) users of oral contraceptives (OCs). **(A)** Never users increased significantly in depressive symptoms in late adolescence, whereas late pill users had a stable trajectory throughout adolescence and early pill users decreased in symptoms after age 16. **(B)** Never, early and late OC users did not show significantly different anxiety symptom trajectories in adolescence. Means are adjusted for sexual debut, romantic relationships, education level, religion, smoking history, alcohol use, and drug use.

Grouping OC use into early and late users resulted in the developmental courses depicted in Figure 2 (see Supplementary Table 4 for model fit, standardized regression coefficients and standard errors of the models). For depressive symptoms, results only showed a significant interaction between age slope 2 and OC use (*p* < 0.001). An increase in depressive symptoms was shown for never users of OCs in late adolescence (> 16 years). Late pill users showed a stable trajectory throughout adolescence and early pill users showed a decrease in depressive symptoms after the age of 16. This effect remained after full adjustment for all covariates (*p* < 0.001).

The growth model for anxiety did not show significant effects of age or OC use, the interaction effect of age slope 2 and OC use had a *p*-value of 0.112. Adjusting for covariates resulted in the same non-significant interaction effect of age slope 2 and OC use (*p* = 0.099).

### Sensitivity Analyses

The sensitivity analysis using a 2-slope model before and after OC onset showed similar effects as the main models comparing never and ever users, see Supplementary Table 1 and Supplementary Figure 1. A significant increase in symptoms for never users in late adolescence was seen for both depressive (*p* = 0.002) and anxiety (*p* = 0.028) symptoms. When considering age of OC onset, the interaction effects were similar (*p* = 0.006 for depression, *p* = 0.086 for anxiety) compared to the models using age to model the slopes. However, in these models the slopes for early and late OC use did not differ from each other (*p* > 0.05) but only from never users (*p* < 0.05).

## Discussion

This study found an altered symptom trajectory of anxiety and depressive symptoms for OC users in late adolescence. Specifically, OC users showed a stable course of depressive and anxiety symptoms throughout adolescence whereas never users showed an increase of internalizing symptoms in late adolescence. When adjusting the developmental trajectories for baseline differences between groups (romantic relationships, sexual debut, educational level, smoking, drinking, and drug use) the effects of OC use remained significant. Furthermore, comparing development before and after OC use resulted in the same effects. That is, no change in symptom development between groups was found before start of OC use, whereas OC users showed a more stable course after OC onset without the increase in symptoms seen on never OC users. Earlier start of OC use even showed a decrease in depressive symptom development after the age of 16, but this effect disappeared when taking the development before and after OC onset into account.

This apparent “protective” role of OC use on internalizing symptoms has been found previously (8, 10–14, 50). In these studies, women using OCs showed lower prevalence of depressive symptoms (12, 13), Major Depressive Disorder (MDD), Generalized Anxiety Disorder (GAD) and Panic Disorder (PD) (10), and even lower risk of past-year suicide attempts (12) compared to non-users. This could be due to a stabilizing effect of OC use on hormonal fluctuations of E and P throughout the menstrual cycle and its co-occurring stabilization of mood variability (51).

The importance of considering relevant confounders when studying OC side effects is shown by these aforementioned studies. They showed that controlling for confounders (including age, BMI, physical activity, chronic disease, number of sexual partners) rendered results non-significant for the protective effects of OCs on psychopathology (8, 10), most likely because OC use in these studies was associated with increased health-promoting behavior (12). In our study, the stabilizing effect of OC use on internalizing symptoms remained after adjustment for relevant risk factors. However, OC use in our study was associated with increased risk behavior for (mental) health problems. Namely, OC users had a lower educational level, were more likely to have had romantic relationships, their sexual debut, a history of smoking, and were heavier drinkers. These are behaviors that are generally associated with an increased risk of internalizing disorders (26, 43, 44). Our results may therefore better fit with the buffer perspective on risky behavior, which suggests that experimenting with alcohol and drugs may not be detrimental *per se* but can be part of developmentally normative activities (52–54). Beyond the covariates regarding risky behavior that were included in this study, OC use may function as an overall indicator of a more outgoing and social lifestyle with OC users representing the typically developing teenagers. As interpersonal difficulties are considered correlates and risk factors for internalizing disorders such as depression (55), the increased socialization of OC users could buffer the increased depression and anxiety symptoms in late adolescence seen in non-users. However, if these groups are to be considered inherently and substantially different, with OC users reflecting the more normative developing adolescents, it could be expected that the groups showed a significant difference in development before OC onset. This was, however, not the case.

Both our raw and modeled data show an overall increase for depressive symptoms in late adolescence, which is in line with previous studies using the same dataset that have shown an increase in internalizing symptoms in late adolescence for boys and girls (56–58). In the study by Nelemans et al. (57), the results for girls showed a larger intercept and less steep incline in depressive symptoms in late adolescence compared to boys. This would imply a more stable course of symptoms over time in girls, which is possibly an effect of higher baseline levels for girls in general or due to the contribution of OC users. The first inclination would be to interpret the finding of a decrease in internalizing symptoms for OC users in late adolescence as protective. Conversely, the increase in symptoms at the end of adolescence could be considered beneficial and part of developmentally appropriate behavior (59), especially because this increase is seen in both boys and girls (57). Adolescence is a developmental period in which internal changes and external challenges result in higher turmoil than in either childhood or adulthood (60). This significant transition period requires adolescents to adapt to the developmental tasks of that period and provides an opportunity to attain psychological autonomy in adulthood (61). As such, OC use may prevent adolescents from learning to express a range of emotions and developing adequate coping strategies.

Our finding that earlier age of OC onset in adolescence strengthens the effect of OCs on depressive symptoms builds on previous research showing that considering age of OC onset is essential when studying mood related side effects of OC use (15–17, 27). However, this effect disappeared when group differences were assessed after OC onset rather than according to age. The previous effect may then be explained by duration of use, as earlier OC users automatically became longer OC users. The effect of age of OC onset may also not be of added value in this study as only adolescent OC users were included, but remains an important factor to be considered when studying adult OC users.

### Strengths, Limitations and Future Directions

In our study we were able to analyze symptom trajectories as a function of OC use over time, which may give more information about effects on mental health than comparing outcomes at any one given time point. The current dataset entailed extensive information regarding adolescent development, which made it possible to include and correct for relevant characteristics predictive of mental health in those years. The methodological challenges when examining OC use on mental health symptoms include problems such as survivor bias and confounding. Our study included information on previous OC use, so we were able to include ever users rather than just current users. Additionally, we were able to correct for most known confounders for both internalizing symptoms and OC use after which the effects remained significant.

Nevertheless, methodological limitations of epidemiological studies like this one are that confounding by unmeasured variables remains a possibility and causality cannot be inferred. For example, our data did not have information on premenstrual symptoms (PMS) or non-contraceptive reasons for using OCs such as acne reduction. The potential protective effect of OCs on mental health may be due to the reduction of hormonal fluctuations, so the positive effects of OC use may be reserved for women who suffer from the hormonal fluctuations of the menstrual cycle and experience a high degree of premenstrual symptoms and related internalizing symptoms that are alleviated by the stabilizing effects of OCs (11). In addition, future research should consider social functioning as a possible buffer in OC users for future psychopathology. Lastly, the contribution of sexual orientation to the increase in internalizing symptoms in never users should be explored. Non-heterosexual girls will be less likely to start OCs and are at increased risk of developing internalizing symptoms in adolescence (62, 63).

It should also be noted that current and past OC use was based on self-report, and was not confirmed by medical reports. Age of onset of OC use could therefore be subject to inaccuracies. Additionally, information on OC use was only assessed at wave 6 when participants were, on average, 18 years old. Participants starting OC use at a later age may have been wrongly classified as “never” users. However, the vast majority of OC users were probably classified correctly at wave 6, because Dutch girls start OC use on average at age 16 (4). Moreover, a wrongful classification would only mean an underestimation of the stabilizing effect seen in OC users. Removing the wrongfully classified OC users from the never user group would also remove the dampening effect seen in OC users from the already increased symptom trajectories seen in never users. In a sensitivity analysis shown in Supplementary Table 5, analyzing only waves 1 through 6 (without 7–9), we found similar results for depression symptoms with significant group differences in late adolescence. Therefore, despite increased differences between groups in standard deviations, the pill effect on depressive symptoms remains apparent in late adolescence. However, the increase in anxiety symptoms in late adolescence for never users rendered insignificant when analyzing only waves 1 through 6. This further showed that the effect of OC use on anxiety symptoms was inconsistent in our sample and depends on the method of analysis (never/ever use, never/early/late use, crude/adjusted, number of waves). Another limitation was the lack of information on type and dose of OC. Considering the different androgenic properties of possible progestins in OCs, different effects on mood are possible and important to consider (7).

After controlling for the aforementioned variables, an effect of OC use on mental health could possibly still be explained by residual confounding by unknown confounders that we could not account for. For example, the confounders could reflect a bigger social buffer effect as mentioned above. At the same time, previous epidemiological studies on OC use and depression have also shown multiple group differences in factors including SES, sexual activity, ethnicity, educational level, BMI, age at menarche, and smoking (8, 15, 16). These recurring group differences point to our sample being representative of the general population.

Our results show the importance of considering age of OC onset when researching mood related effects. However, OC onset data were not available for 41 participants, resulting in more missing data in these analyses compared to the never/ever analyses. Therefore, the results of this analysis have to be interpreted with caution due to lower power and could potentially explain the lack of significant findings with respect to age of OC use onset and anxiety symptom development.

## Conclusion

In conclusion, this study underlines the importance of considering OC use in the development of internalizing symptoms from adolescence into adulthood and adds to research showing that OC affects mental health. Our results suggest that OC users show a stable trajectory of anxiety and depressive symptoms throughout adolescence whereas girls who do not use OCs show an increase in internalizing symptoms in late adolescence, even after adjusting for confounders. The stabilizing trajectory in OC users is surprising given that OC users reported more health risk behaviors, but it may reflect a group of typically developing girls. The question is whether this altered symptom trajectory in OC users can be considered as a protective effect of OC use on psychopathology and which developmental pattern can be seen as the normative development of internalizing symptoms in adolescent girls. The answer may lie in future studies predicting adaptive outcomes in later life using the separate developmental trajectories of depressive and anxiety symptoms based on OC use. Given the high number of adolescent girls that start OCs in adolescence (3, 4), additional research is needed to improve our understanding of the long-term consequences of OC use on mental health.

## Data Availability Statement

Publicly available datasets were analyzed in this study. This data can be found at: https://doi.org/10.17026/dans-zrb-v5wp (For Waves 1-7, data used for this study are available on request to the corresponding author).

## Ethics Statement

The studies involving human participants were reviewed and approved by Medical Ethical Committees of the Utrecht Medical Center and VU University Medical Center, Netherlands, and the Ethical Committee of the Faculty of Social Science of the Utrecht University, Netherlands. Written informed consent to participate in this study was provided by the participants' legal guardian/next of kin.

## Author Contributions

AD: formal analysis, writing—original draft, and visualization. SB, SN, EM, IE, JB, and WM: conceptualization and writing—review and editing. LG: formal analysis, conceptualization, writing—original draft, and supervision. All authors contributed to the article and approved the submitted version.

## Funding

Data of the RADAR (Research on Adolescent Development and Relationships) study were used. RADAR has been financially supported by main grants from the Netherlands Organization for Scientific Research (GB-MAGW 480-03-005 and GB-MAGW 480-08-006), Stichting Achmea Slachtoffer en Samenleving (SASS), the Netherlands Organization for Scientific Research to the Consortium Individual Development (CID; 024.001.003), a grant of the European Research Council (ERC-2017-CoG - 773023 INTRANSITION), and various other grants from the Netherlands Organization for Scientific Research, VU University Amsterdam, and Utrecht University. IE was funded by a Vici innovational research grant (453–15–005) from the Netherlands Organization for Scientific Research. The funders had no role in the study design, data collection and analysis, decision to publish, or preparation of the manuscript.

## Conflict of Interest

The authors declare that the research was conducted in the absence of any commercial or financial relationships that could be construed as a potential conflict of interest.

## Publisher's Note

All claims expressed in this article are solely those of the authors and do not necessarily represent those of their affiliated organizations, or those of the publisher, the editors and the reviewers. Any product that may be evaluated in this article, or claim that may be made by its manufacturer, is not guaranteed or endorsed by the publisher.
