# Supplementary material for: Stable Anxiety and Depression Trajectories in Late Adolescence for Oral Contraceptive Users
Source: Front Psychiatry. 2022 May 23;13:799470. doi: 10.3389/fpsyt.2022.799470 (PMC9168124; doi:10.3389/fpsyt.2022.799470)
Supplement: Supplementary file 1 [file Table_1.pdf]

Supplementary Material

Supplementary Table 1. Model fit**,** standardized regression coefficients and standard errors of fixed and random effects as predictors of development of depressive and anxiety symptoms with never and ever users, and never, early and late users of oral contraceptives modeled by OC onset**.**

Parameters	Estimate	SE	χ^2	df	AIC	BIC
**Depression [never/ever]**						
Intercept	39.85	1.73	502.77	6	4941.4	4961.8
Time1	0.68	0.44				
Time2	2.30**	0.56				
OC use	1.05	2.38				
OC use*time1	-0.52	0.63				
OC use*time2	-2.18*	0.70				
**Depression [never/early/late]**						
Intercept	39.84	1.73	501.66	6	4931.5	4951.9
Time1	0.68	0.44				
Time2	2.29	0.56				
OC use early	2.26	2.90				
OC use late	-0.28	2.91				
OC use early* time1	0.39	1.07				
OC use late* time1	-0.88	0.69				
OC use early*time2	-2.33*	0.73				
OC use late* time2	-1.75	1.36				
**Anxiety [never/ever]**						
Intercept	52.15	1.58	479.56	6	4763.5	4783.9
Time1	-0.03	0.49				
Time2	1.34	0.60				
OC use	0.89	2.20				
OC use*time1	0.28	0.70				
OC use*time2	-1.77*	0.81				
**Anxiety [never/early/late]**						
Intercept	52.15	1.59	479.58	6	4753.8	4774.2
Time1	-0.03	0.49				
Time2	1.34	0.60				
OC use early	0.32	2.69				
OC use late	1.47	2.71				
OC use early* time1	1.05	1.15				
OC use late*time2	0.19	0.78				
OC use early*time1	-1.73	0.86				
OC use late* time2	-2.11	1.41				

*p < 0.05, **p <0.001; Time1 refers to measures predating OC start, Time2 to measures after OC start; SE: standard error; df: degrees of freedom. AIC: Akaike Information Criterion; BIC: Bayesian Information Criterion.

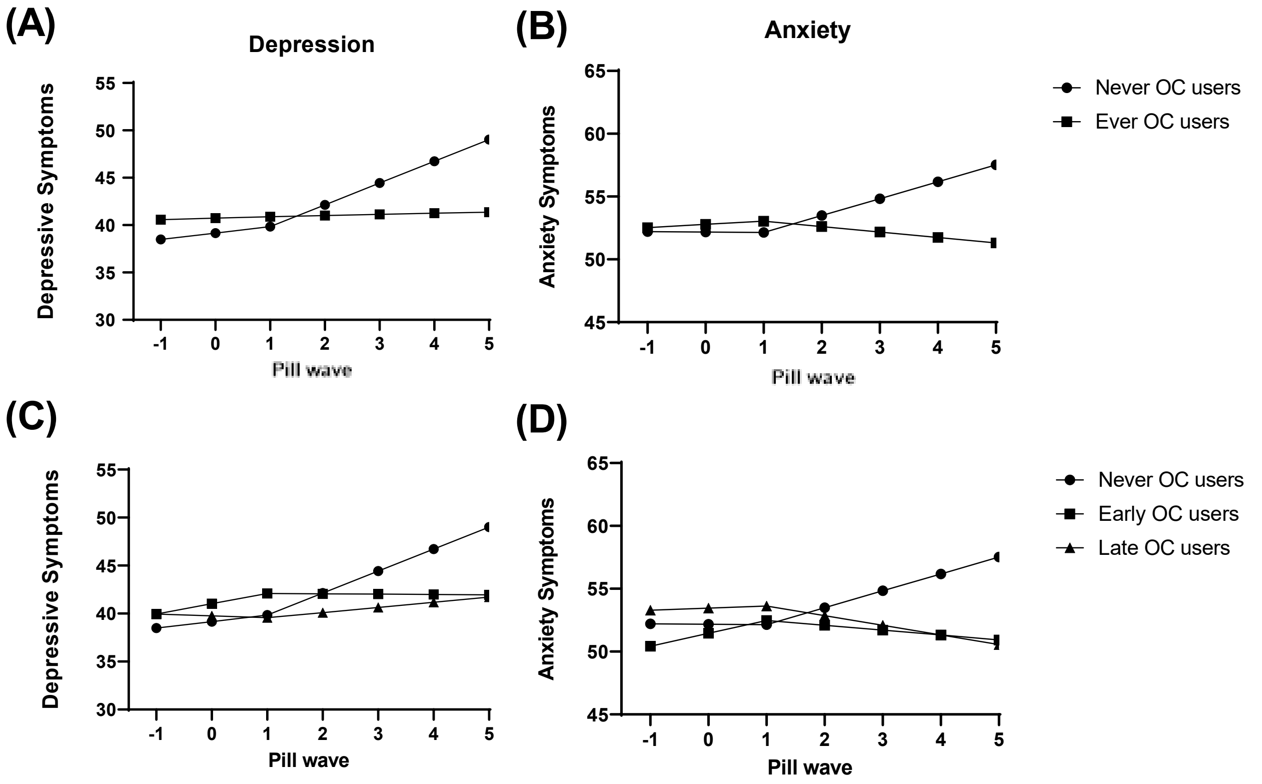

Supplementary Figure 1. Oral Contraceptive (OC) use, age of onset, and modeled anxiety and depression trajectories based on OC onset. Growth curve models for the depressive and anxiety symptoms for never and ever users, and never, early (<15 years), and late (≥15 years) users of oral contraceptives (OCs); pill wave refers to measurements taken before (-1,0) and after (>0) OC onset.

Supplementary Table 2. Correlations between study sample characteristics and depression and anxiety symptoms.

**Characteristic**	**Depression W6**		**Anxiety W6**	
	Pearson's r	P	Pearson's r	P
Age, mean (SD)	-0.13	0.080	-0.11	0.140
Age at menarche, mean (SD)	0.08	0.300	0.07	0.401
Age at sexual debut, mean (SD)	-0.17	0.096	-0.05	0.627
Sexual debut, N(%)	-0.08	0.312	-0.08	0.286
Romantic Relationships, N(%)	-0.09	0.276	-0.10	0.189
Education, mean (SD)	0.05	0.562	0.09	0.253
Low Family SES, N(%)	0.05	0.468	-0.02	0.781
Religious, N(%)	-0.01	0.856	0.02	0.821
Smoking history, N(%)	-0.01	0.902	-0.02	0.839
Alcohol Use, mean (SD)	-0.16	0.030	-0.16	0.038
Drug Use, N(%)	0.09	0.232	0.03	0.659
Childhood Trauma, mean (SD)	0.43	< 0.001	0.31	0.012
Neuroticism[a], mean (SD)	-0.28	< 0.001	-0.33	< 0.001

[a] Scores reflect emotional stability with lower scores meaning higher levels of neuroticism

Supplementary Table 3. Correlations between anxiety and depressive symptoms for each wave.

**Wave**	Pearson's r	P
Age 13 (W1)	0.67	< 0.001
Age 14 (W2)	0.72	< 0.001
Age 15 (W3)	0.77	< 0.001
Age 16 (W4)	0.78	< 0.001
Age 17 (W5)	0.79	< 0.001
Age 18 (W6)	0.81	< 0.001
Age 20 (W7)	0.79	< 0.001
Age 22 (W8)	0.83	< 0.001
Age 24 (W9)	0.73	< 0.001

Supplementary Table 4. Model fit**,** standardized regression coefficients and standard errors of fixed and random effects as predictors of development of depressive and anxiety symptoms with never, early and late users of oral contraceptives**.**

Parameters	Estimate	SE	χ^2	df	AIC	BIC
**Depression - crude**						
Intercept	38.12	1.81	721.83	6	6880.9	6901.3
Age1	0.23	0.45				
Age2	1.10	0.25				
OC use early	4.60	3.09				
OC use late	2.19	2.79				
OC use early*age1	0.83	0.80				
OC use late*age1	-0.56	0.71				
OC use early*age2	-1.77**	0.54				
OC use late*age2	-1.10*	0.40				
**Depression - adjusted**						
Intercept	35.02	4.22	619.86	6	6204.0	6223.5
Age1	0.29	0.48				
Age2	1.16	0.26				
OC use early	6.54	3.61				
OC use late	4.45	3.31				
OC use early*age1	0.65	0.86				
OC use late*age1	-0.35	0.76				
OC use early*age2	-1.80**	0.54				
OC use late*age2	-1.07*	0.41				
**Anxiety - crude**						
Intercept	38.12	1.81	721.83	6	6880.9	6901.3
Age1	0.23	0.45				
Age2	1.10	0.25				
OC use early	4.60	3.09				
OC use late	2.19	2.79				
OC use early*age1	0.83	0.80				
OC use late*age1	-0.56	0.71				
OC use early*age2	-1.77*	0.54				
OC use late*age2	-1.10**	0.40				
**Anxiety - adjusted**						
Intercept	55.54	4.04	581.86	6	5734.5	5753.9
Age1	-0.58	0.52				
Age2	0.58	0.24				
OC use early	2.31	3.29				
OC use late	2.65	3.02				
OC use early*age1	1.41	0.96				
OC use late*age1	-0.14	0.82				
OC use early*age2	-1.08	0.51				
OC use late*age2	-0.41	0.40				

*p < 0.05, **p < 0.001; Adjusted models are corrected for romantic relationships, sexual debut, educational level, religion, smoking history, alcohol use and drug use; SE: standard error; df: degrees of freedom. AIC: Akaike Information Criterion; BIC: Bayesian Information Criterion.

Supplementary Table 5. Comparison of standardized regression coefficients and standard errors of fixed and random effects as predictors of development of depressive and anxiety symptoms using waves 1-9 or waves 1-6.

**Parameters**	**Wave 1-9**		**Wave 1-6**	
	**Estimate**	**SE**	**Estimate**	**SE**
**Depression - adjusted**				
Intercept	39.09	3.68	40.18	3.83
Age1	0.27	0.49	0.27	0.54
Age2	1.20*	0.26	1.26	0.74
OC use	4.54	2.56	4.81	2.69
OC use*age1	-0.09	0.61	0.06	0.67
OC use*age2	-1.30**	0.34	-1.95*	0.91
Romantic Relationships	-4.23	2.53	-4.56	2.63
Sexual Debut	-0.06	2.32	0.29	2.40
Educational Level	0.51	0.34	0.44	0.35
Religion	-1.48	1.67	-1.73	1.72
Alcohol Use	-3.06*	1.05	-3.34*	1.09
Smoking	4.20*	1.85	3.79*	1.92
Drug Use	5.18*	2.05	5.34*	2.12
**Anxiety - adjusted**				
Intercept	55.11	3.42	55.86	3.51
Age1	-0.60	0.53	-0.54	0.54
Age2	0.59	0.24	0.39	0.66
OC use	2.69	2.32	3.04	2.42
OC use*age1	0.52	0.68	0.44	0.69
OC use*age2	-0.74*	0.32	-0.65	0.82
Romantic Relationships	-4.66	2.38	-5.50*	2.44
Sexual Debut	-0.05	2.20	-0.16	2.25
Educational Level	0.21	0.32	0.27	0.33
Religion	-0.72	1.58	-0.69	1.61
Alcohol Use	-3.10*	0.99	-3.51**	1.00
Smoking	2.39	1.75	2.64	1.79
Drug Use	5.93*	1.94	5.47*	1.98

*p < 0.05, **p < 0.001; Adjusted models are corrected for educational level, religion, smoking history, alcohol use and drug use; SE: standard error; df: degrees of freedom. AIC: Akaike Information Criterion; BIC: Bayesian Information Criterion.
